# Roles of healthcare professionals in the management of chronic gastrointestinal diseases with a focus on primary care: A systematic review

**DOI:** 10.1002/jgh3.12235

**Published:** 2019-08-27

**Authors:** Sharmila S Prasad, Michael Potter, Simon Keely, Nicholas J Talley, Marjorie M Walker, Therése Kairuz

**Affiliations:** ^1^ Faculty of Health and Medicine, School of Biomedical Science and Pharmacy University of Newcastle Callaghan New South Wales Australia; ^2^ Priority Research Centre, Digestive Health and Neurogastroenterology University of Newcastle New Lambton Heights New South Wales Australia; ^3^ Faculty of Health and Medicine, School of Medicine and Public Health University of Newcastle New Lambton Heights New South Wales Australia

**Keywords:** gastrointestinal, healthcare professional, inflammatory bowel disease, primary care

## Abstract

**Background and aim:**

Inflammatory bowel disease (IBD) refers to a group of complex and chronic conditions that requires long‐term care delivered by a group of healthcare professionals through a multidisciplinary care model. We conducted a systematic review to examine and understand the role of healthcare professionals in the primary care management of IBD, and identify the gaps in IBD management that could be filled by primary care providers such as general practitioners (GPs) and pharmacists.

**Methods:**

The search strategy retrieved published studies from five databases, and eligible articles were assessed for quality. A gray literature search of the websites of organizations was also undertaken.

**Results:**

Twenty‐one studies were included, of which 19 were peer‐reviewed research articles and two reports were from organizational bodies. Although studies have shown the roles of GPs, pharmacists, dietitians, and psychologists in IBD management, nurses and gastroenterologists were the key drivers delivering specialized care to IBD patients. Many key services are accessible only for hospital inpatients (tertiary care) or through outpatient clinics (secondary care) with an absence of a multidisciplinary approach including GPs and pharmacists.

**Conclusion:**

Gastroenterologists and nurses have an important role in the delivery of care to patients with chronic gastrointestinal diseases including IBD, coeliac disease, irritable bowel syndrome, and functional dyspepsia. The role of nurses includes provision of specialized care to IBD patients, as well as supportive care such as education, monitoring of therapy, and ongoing assistance. The available evidence shows many opportunities for primary care providers to play a more active role in the management of IBD patients.

## Graphical abstract

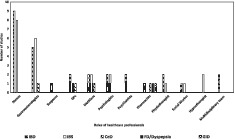

Although the review showed the roles of general practitioners (GPs), pharmacists, dietitians, and psychologists in inflammatory bowel disease (IBD) management, nurses and gastroenterologists were the key drivers delivering specialized care to IBD patients. Many key services are accessible only for hospital inpatients (tertiary care) or through outpatient clinics (secondary care) with an absence of a multidisciplinary approach including GPs and pharmacists. The available evidence shows opportunities for primary care providers to play a more active role in the management of IBD patients.

## Introduction

The global burden of gastrointestinal disease is increasing and has been estimated at 6–60 billion cases annually.^1^ Gastrointestinal diseases range from those with minor self‐limiting symptoms to those with chronic debilitating symptoms such as abdominal pain, weight loss, fatigue and tiredness, and changes in bowel habits.^2^, ^3^, ^4^ Inflammatory bowel diseases (IBDs) are chronic diseases of the gastrointestinal tract that are among the most burdensome and difficult to manage even though the prevalence is only 0.4% in the general population.^5^, ^6^ IBD can be classified into two major types,^3^, ^7^, ^8^, ^9^ Crohn's disease (CD), which is incurable and is associated with an increased mortality risk, and ulcerative colitis (UC) that can only be cured with total colectomy.^2^, ^3^, ^4^, ^10^ In addition to the overall impact on the quality of life, IBD affecting the colon is also an important risk factor for colorectal cancer.^3^, ^8^, ^9^

IBD is among the top five most expensive gastrointestinal diseases to treat^11^, ^12^ and incurs considerable social costs and reduces patients' quality of life.^8^ It has been referred to as “an emerging global disease” of the developed world,^8^ but recent literature has also shown an increasing incidence in developing nations as they have become more industrialized.^13^, ^14^, ^15^ Because of its high morbidity and increasing prevalence and costs, IBD management is an issue of considerable concern.^4^, ^8^, ^13^, ^16^ Onset usually occurs in early adulthood and thus IBD requires lifelong management.^3^, ^7^, ^8^, ^9^ When these factors are considered, IBD represents a disproportionately burdensome and costly disease relative to disease prevalence, and as the incidence and burden of IBD continues to rise, improved IBD management is essential.^6^, ^17^, ^18^

The aim of IBD management is to induce and maintain remission and ultimately improve the patients' quality of life while reducing burden of disease. Furthermore, IBD is a complex condition that often requires a multidisciplinary team approach to achieve optimal quality care.^7^, ^8^, ^19^, ^20^, ^21^ In other chronic conditions such as diabetes and asthma, multidisciplinary team approaches have led to marked improvements in multiple aspects of chronic disease management. Diabetic patients now have improved glycated hemoglobin (HbA1c) levels, improved medication adherence/compliance, and access to education and support through collaboration with specialists, general practitioners (GPs), pharmacists, dietitians, physiotherapists, and other healthcare professionals.^22^, ^23^, ^24^, ^25^, ^26^, ^27^, ^28^, ^29^ However, the role of healthcare professionals in the primary care management of IBD management is not yet clearly defined. Although there is increasing research in the field of IBD, much of it relates to specialist management in secondary/tertiary care settings with limited reports, highlighting the role of healthcare professionals in the provision of IBD care in the primary setting.^30^ The purpose of this systematic review is therefore to examine the roles of healthcare professionals involved in the management of IBD in contrast to their roles in the management of other chronic gastrointestinal diseases in the primary care setting. A secondary aim is to identify the gaps in primary care IBD management and to explore potential roles for allied/PCPs (primary care physicians) to deliver care to IBD patients.

## Materials and methods

This systematic review follows the Preferred Reporting Items for Systematic Reviews (PRISMA) guidelines^31^, ^32^ as outlined later.

### *Search strategy and study eligibility*

A systematic search of five bibliographic databases (Medline, Embase, CINAHL, PsycINFO, and Scopus) and of the gray literature was conducted to identify studies relating to the roles of healthcare professionals in IBD. A separate search in the Cochrane database was performed to identify systematic reviews with the same content.

Review articles, editorials, notes, commentaries, non‐original studies, and studies focusing only on secondary or tertiary care were excluded. The inclusion criteria were articles published in English with a date restriction of 1970 onwards, and experimental and observational studies that reported inventions describing any services delivered by medical and/or allied healthcare professionals related to the management of patients with gastrointestinal diseases in a primary care setting (see Table S1, Supporting information). The study design included randomized controlled trials (RCT), non‐randomized control trials, and cohort and case–control studies.

There is a paucity of published literature about the primary care management of IBD, and the initial search (for IBD) resulted only in articles relating to specialist care by gastroenterologists and hospital nurses in secondary and tertiary settings. Therefore, the search terms were broadened to include chronic gastrointestinal diseases. Although the needs of individuals and treatment options in IBD are different from other chronic gastrointestinal diseases, the management is somewhat similar because it also involves a multidisciplinary team approach; the revised strategy also provided a useful platform to examine the roles of other healthcare professionals.

The key search terms used the following three Medical Subject Headings (MeSH): disease (gastrointestinal diseases, IBD, coeliac disease (CeD), irritable bowel syndrome (IBS), and dyspepsia), setting (primary care/community), and profession (healthcare professionals—nurses, gastroenterologists, GPs, dietitians, and pharmacists). To conduct the search in the gray literature, the websites of professional organizations/societies (see Table S2) were assessed, and a manual bibliographic search of the conference abstracts arising from the systematic search was performed. Furthermore, a general search using the Google search engine was performed, the first 100 results of which were then reviewed.

All duplicate articles were removed. The overall screening for title, abstract, and full text was completed by two independent reviewers (SP and MP) who assessed the eligibility of each article based on the inclusion and exclusion criteria. Disagreements were resolved via consensus.

### *Quality assessment*

Because of the wide range of study designs, an adapted version of the quality assessment criteria, as defined by Nagpal *et al*.^33^ and Turner‐Stokes *et al*.,^34^ as well as a 3‐point ordinal scoring scale, were developed. The same two reviewers independently evaluated the quality of each study. Cohen's kappa^35^, ^36^ was used to indicate the degree of agreement between the two reviewers regarding the quality of this review. Studies with scores of less than 7/20 were deemed to be of poor quality and were therefore excluded.

### *Data extraction*

The study characteristics of the included articles were systematically entered into a customized spreadsheet (see Table S3) and included bibliographic reference (first author, year, and reference number), study design/methodology, study population (healthcare professionals), key findings (results and conclusion), level of evidence, and comments on the document. The extraction process was performed by two reviewers (SP and MP).

## Results

### *Study selection*

**Figure 1:**
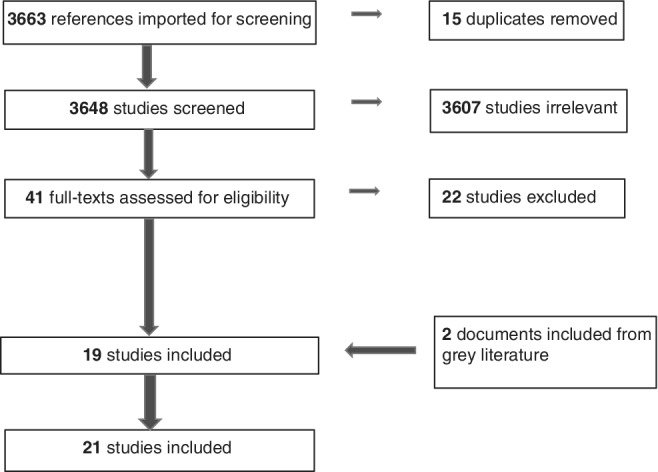
PRISMA flow diagram that describes the process and results of the systematic search undertaken.

In all 3663 citations were identified from five databases, and 41 potential studies were selected for full‐text screening (Fig. 1). Of the selected studies, twenty‐two did not meet the inclusion criteria and the remaining studies (*n* = 19) were appraised for quality.^37^, ^38^, ^39^, ^40^, ^41^, ^42^, ^43^, ^44^, ^45^, ^46^, ^47^, ^48^, ^49^, ^50^, ^51^, ^52^, ^53^, ^54^, ^55^, ^56^ In the search for gray literature, six potential documents were found in the web pages of two organizations, namely Crohn's and Colitis Australia (three documents)^7^, ^8^, ^57^ and Crohn's and Colitis UK (three documents)^58^, ^59^, ^60^; two of these documents were relevant and met the study criteria.^7^, ^58^ Thus, a total of 21 articles provided relevant information on the roles of healthcare professionals in gastrointestinal diseases.^7^, ^37^, ^38^, ^39^, ^40^, ^41^, ^42^, ^43^, ^44^, ^45^, ^46^, ^47^, ^48^, ^49^, ^50^, ^51^, ^52^, ^53^, ^54^, ^55^, ^58^

### *Assessment of quality*

During quality appraisal, the maximum score achieved for the studies included in our review was 19 out of a possible 20 (deemed high quality) and there were 12 articles included in this category,^7^, ^37^, ^38^, ^44^, ^47^, ^49^, ^50^, ^51^, ^52^, ^53^, ^56^, ^58^ of which nine articles (with scores of 7–13) were of medium quality. A Cohen's kappa index score of 0.8 demonstrated a degree of substantial agreement between the two reviewers (SP and MP).

### *Description of studies*

**Figure 2:**
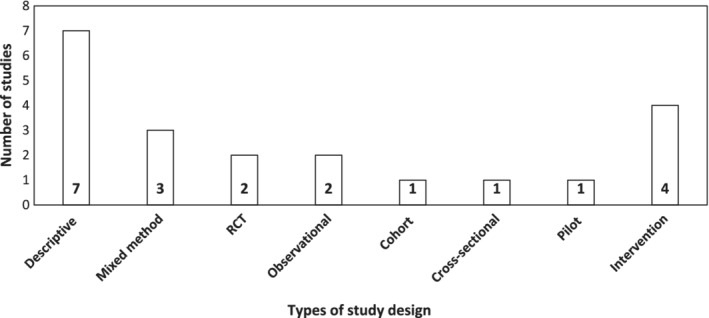
Breakdown of the number of studies associated with each study design type. This shows methodological heterogeneity of the included studies which used qualitative and quantitative analyses.

With regard to the study design or methodology, just under one‐third of the studies used qualitative methodology^41^, ^43^, ^45^, ^48^, ^53^, ^54^ and most used quantitative methodology,^37^, ^38^, ^40^, ^42^, ^43^, ^44^, ^45^, ^46^, ^47^, ^48^, ^49^, ^50^, ^51^, ^52^, ^53^, ^55^, ^56^ including randomized control trials,^44^, ^50^ cohort study design,^52^ cross‐sectional study design,^48^ non‐randomized intervention study design,^38^, ^40^, ^45^, ^46^, ^49^ pilot study design,^55^ and studies with mixed methodology,^43^, ^45^, ^53^ which also included observational study design examining quality standards^7^, ^58^ (Fig. 2).

Of the included studies (*n* = 21), the majority related to IBD (*n* = 11),^7^, ^41^, ^42^, ^43^, ^46^, ^48^, ^51^, ^54^, ^55^, ^56^, ^58^ six studies were of IBS,^38^, ^40^, ^42^, ^44^, ^49^, ^50^ two investigated CeD,^37^, ^53^ one study examined dyspepsia (including functional dyspepsia [FD]),^45^ two studies were associated with the symptoms and management of gastrointestinal diseases in general,^47^, ^52^ and one study assessed more than one gastrointestinal disease.^42^ The setting and research focus of the studies typically came from European countries such as the United Kingdom, Sweden, Netherlands, Germany, Belgium, Denmark, and many more, followed by other westernized countries such as Australia, Canada, and the United States of America involving urban (including metropolitan) and rural locations.

### *Gaps in care is a research focus specific in IBD among chronic gastrointestinal diseases*

**Figure 3:**
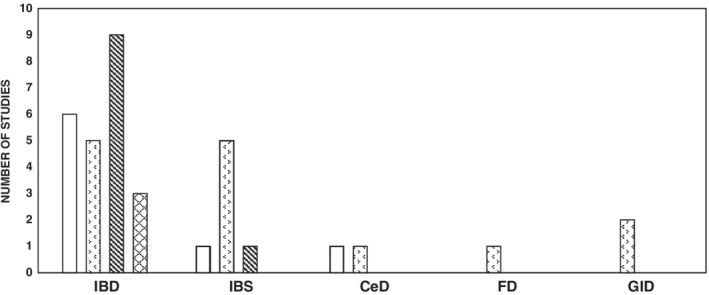
Study themes associated with managing gastrointestinal (GI) diseases. IBD, inflammatory bowel disease; IBS, irritable bowel syndrome; CeD, coeliac disease. 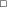, Perception; 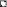, disease management; 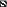, gaps in care; 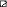, burden of disease.

The included studies were classified according to the following four key themes: perception (9 studies),^37^, ^41^, ^42^, ^43^, ^48^, ^51^, ^54^, ^55^, ^56^ disease management (11 studies),^38^, ^40^, ^44^, ^45^, ^46^, ^47^, ^49^, ^50^, ^52^, ^53^, ^56^ gaps in care (9 studies),^41^, ^42^, ^43^, ^48^, ^51^, ^54^, ^55^ and burden of disease (three studies).^7^, ^46^, ^58^ Almost half of the included studies (*n* = 9) related to more than one theme, such as perception, gaps in care, and disease management^41^, ^42^, ^43^, ^46^, ^48^, ^51^, ^54^, ^55^, ^56^ and appear to be more evident in IBD than other gastrointestinal diseases, such as CeD and dyspepsia (Fig. 3).

Perception studies focused on understanding the views and opinions of key stakeholders including gastroenterologists, nurses, patients, and other healthcare professionals (i.e., psychologists, dietitians, GPs, psychiatrists, pharmacists, and physiotherapists) whose primary goal was managing various aspects of IBD.^41^, ^42^, ^43^, ^48^, ^51^, ^54^, ^55^, ^56^ Studies examined patients' willingness/concerns regarding an IBD nurse telephone follow‐up service, the long‐term follow‐up method in the management of CeD, healthcare professionals' views on improving therapeutic adherence in UC, their perceptions of fatigue and its impact on patients with IBD, and nurses' views on the provision of IBD services.^37^, ^41^, ^46^, ^54^, ^56^ Disease management studies evaluated the effectiveness of interventions in the management of a variety of chronic gastrointestinal diseases such as IBD, IBS, CeD, and dyspepsia (including FD).^38^, ^40^, ^44^, ^45^, ^46^, ^47^, ^49^, ^50^, ^52^, ^53^, ^56^ Studies explored self‐management, the effectiveness of nurse‐led interventions, adherence to pharmacist advice, and the effectiveness of pharmacist/pharmacy‐led testing in a targeted case‐finding service. Studies investigating gaps in care addressed the lack of or variation in available care in the management of IBD, including gaps in communication (between primary care and specialized care providers), knowledge gaps among healthcare professionals, and the variation in the provision of IBD management and care.^41^, ^42^, ^43^, ^48^, ^51^, ^54^, ^55^ Finally, burden of disease studies evaluated the impact of gaps in and the delivery of IBD care that affect the overall management of IBD.^7^, ^46^, ^58^

Although all four themes were present in IBD, it was not the case for the other chronic gastrointestinal diseases, namely IBS, CeD, and FD/dyspepsia. IBS was evident in three themes, namely perception, disease management, and gaps in care, whereas CeD was present in perception and disease management, and dyspepsia (including FD) was only identified in disease management. Disease management was the only theme present in all four of the chronic gastrointestinal diseases. Burden of disease was only identified in IBD but was not a focus in CeD, FD, and IBS. Approximately 41% (*n* = 9) of IBD research studies involving healthcare professionals investigated gaps in care, and this appeared to be a major research topic. In contrast, IBS had only one study looking at gaps in care and CeD and FD had no studies; the majority of the focus for these conditions was on disease management. This may reflect the fact that IBS, FD, and CeD have more focus on the primary care of the patients than IBD, which is largely managed by specialists and nurses. This can lead to gaps in care, and indeed all of the reviewed studies showed that gaps in care was the predominant theme in IBD research literature.^7^, ^41^, ^42^, ^43^, ^48^, ^51^, ^54^, ^55^, ^58^ This indicates that there are gaps in care in IBD management, which is not evident in other chronic gastrointestinal diseases.

### *Gastroenterologists and nurses are the predominant healthcare professionals in IBD management*

**Figure 4:**
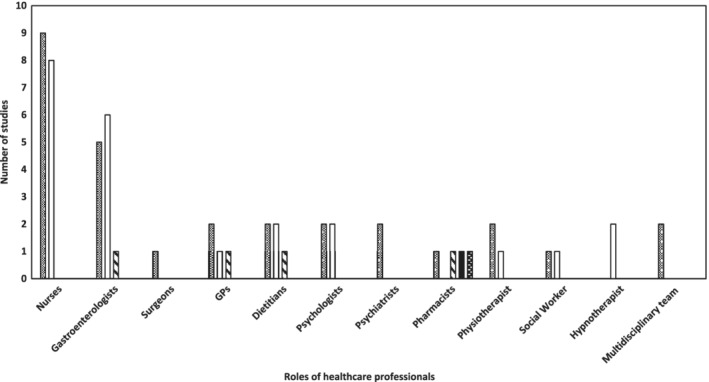
Healthcare professionals involved in the management of chronic gastrointestinal diseases. IBD, inflammatory bowel disease; IBS, irritable bowel syndrome; CeD, coeliac disease. 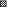, IBD; 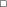, IBS; 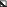, CeD; 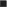, FD/dyspepsia; 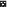, GID.

Gastroenterologists and nurses were identified as the key care providers in IBD and IBS. The most common roles of nurses were categorized into four types of management (Table 1). While eight studies were associated with only a single group of healthcare professionals (four were associated with nurses^46^, ^51^, ^55^, ^56^ and four included pharmacists^45^, ^47^, ^52^, ^53^), the remaining 13 studies were associated with more than one healthcare professional.^37^, ^38^, ^40^, ^41^, ^42^, ^43^, ^44^, ^48^, ^49^, ^50^, ^54^ Twelve studies (24%) investigated the role of nurses,^38^, ^40^, ^41^, ^42^, ^43^, ^46^, ^48^, ^49^, ^51^, ^54^, ^55^, ^56^ 10 (20%) were associated with gastroenterologists^37^, ^38^, ^41^, ^42^, ^43^, ^44^, ^48^, ^49^, ^50^, ^54^; 5 (10%) studies involved pharmacists,^41^, ^45^, ^47^, ^52^, ^53^ dietitians,^37^, ^38^, ^41^, ^48^, ^49^ and other healthcare professionals (surgeons,^48^ social workers,^38^, ^41^ and hypnotherapists^40^, ^50^); while 8% (*n* = 4) involved psychologists^41^, ^44^, ^48^, ^49^ as well as GPs,^37^, ^41^, ^50^, ^54^ 6% (*n* = 3)^41^, ^48^, ^49^ included physiotherapists, and 4% (*n* = 2) involved psychiatrists^41^, ^48^ (Fig. 4). In the management of IBD alone, two studies involved multidisciplinary teams.

**Table 1:** Summary of the roles of nurses identified in the review

Type of management/roles	Tasks involved
Patients/systems management^7^, ^38^, ^40^, ^41^, ^42^, ^43^, ^46^, ^48^, ^49^, ^51^, ^54^, ^55^, ^56^, ^61^, ^62^, ^63^	Manage newly diagnosed patients, triage primary care referrals, and liaising with multi‐disciplinary team
Educational/supportive management^7^, ^38^, ^40^, ^41^, ^42^, ^43^, ^46^, ^48^, ^49^, ^51^, ^54^, ^55^, ^56^, ^61^, ^62^, ^63^	Provide education and counseling on disease and drugs, run helplines, and provide inpatient support
Clinical management^7^, ^38^, ^40^, ^41^, ^42^, ^43^, ^46^, ^48^, ^49^, ^51^, ^54^, ^55^, ^56^, ^61^, ^62^, ^63^	Involving patient assessment, monitoring response to treatment, delivering treatments and services, for example, nurse‐led hypnotherapy, anemia screening by nurse‐led service, and administer and monitor biologics
Research and advocacy^7^, ^46^, ^63^	Conducting clinical IBD research

Overall, the studies reported that 10 health professionals were involved in IBD management compared to other chronic gastrointestinal diseases, for which the statistics were eight health professionals in IBS, four in CeD, and one in dyspepsia/FD (Table 2). Those health professionals frequently contributing to IBD and IBS management included gastroenterologists and nurses followed by less commonly listed health professionals such as dietitians, psychologists, GPs, and physiotherapists, whereas the roles of pharmacists (hospital), surgeons, and psychiaristists were identified in IBD and social workers and hypnotherapists in IBS. In constrast to IBD and IBS, CeD showed an engagement of fewer health professionals such as gastroenterologists, dietitians, GPs, and pharmacists. Pharmacists were identified as the only non‐specialist health professionals involved in IBD, CeD, dyspepsia (including FD), and gastrointestinal diseases in general. Gastroenterologists, dietitians (non‐specialist), and GPs (non‐specialist) were commonly identified as health professionals involved in the management of three chronic gastrointestinal diseases, namely IBD, IBS, and CeD.

**Table 2:** List of identified roles of healthcare professionals in gastrointestinal diseases

Healthcare professionals	Number of studies	GI diseases
Nurses	12	IBD; IBS
Gastroenterologists	10	IBD; IBS; CeD
Dietitians	5	IBD; IBS; CeD
Pharmacists	5	IBD; CeD, dyspepsia
General Practitioners (GP)	4	IBD; IBS; CeD
Psychologists	4	IBD; IBS
Physiotherapists	3	IBD; IBS
Psychiatrists	2	IBD
Social workers	2	IBD; IBS
Hypnotherapists	2	IBS
Multidisciplinary team	2	IBD
Surgeons	1	IBD

CeD, coeliac disease; GP, general practitioners; IBD, inflammatory bowel disease; IBS, irritable bowel syndrome.

## Discussion

The aim of this review was to understand the roles of healthcare professionals in the management of IBD, while also identifying potential gaps in IBD management. Our study highlights the reliance on nursing and gastroenterologist's specialist care in the management of IBD. While this trend was similar to care utilized by IBS patients, studies in IBD tended to emphasize a research focus on gaps in care concerning the management of patients; a similar research focus was not evident in IBS or other chronic gastrointestinal diseases. Although there was limited literature pertaining to primary healthcare providers in the management of IBD overall, these findings may reflect that IBD management, in practice, does not effectively utilize primary healthcare providers, while management of other chronic gastrointestinal conditions has greater reliance on primary care.^48^, ^64^, ^65^ These findings provide important insights into both the impact of IBD on patients and the approaches to IBD management by healthcare professionals, indicating that the current primary care management of IBD could be optimized and such a strategy could effectively address perceived gaps in care in IBD management.

A key finding of this study was the extensive involvement of nurses and gastroenterologists as the key providers in the care of IBD patients as well as those with IBS. This is consistent with other published literatures, demonstrating that nurses are already known to be integral in the management of IBD patients.^66^, ^67^, ^68^ Common roles for nurses providing care to IBD patients relate to educational/supportive management, patients and systems management, clinical management and research, and advocacy.^66^, ^68^, ^69^ Nurses involved in IBD care also have roles as “IBD educators” to ensure the delivery of IBD care associated with hospital services, including assistance with pre‐operative preparation and practical stoma training, nurse‐led colorectal clinics, and ongoing education and stoma‐related support.^59^, ^66^, ^67^ There was, however, only limited engagement from other healthcare professionals such as GPs, dietitians, psychologists, and pharmacists.^7^, ^48^, ^58^ This demonstrates that IBD is predominantly a specialist‐managed condition. In contrast to other chronic gastrointestinal diseases, IBD, because of its complexity, requires ongoing involvement from gastroenterologists during the course of the disease, that is, lifelong specialist management of IBD patients. While it is important to have specialist care, it nevertheless places a disproportionate burden on gastroenterologists, who often have to manage *all* aspects of the disease. Some examples of these are managing adherence to maintenance therapy, giving advice regarding smoking cessation, giving advice on vaccinations and travel, and screening for colorectal cancer and osteoporosis.^8^, ^70^ Given that chronic gastrointestinal diseases such as IBD, IBS, dyspepsia, and CeD are all very different diseases in presentation, severity, treatment, and patients also having very different needs, the overall goal for managing these diseases remains the same: Through multidisciplinary team approaches, to achieve disease control, maintenance of control, improved quality of life for patients, and a reduced burden of disease. However, in IBS, CeD, and dyspepsia, much of the management occurs in primary care mostly involving PCPs. In an organic disease such as CeD, although patients are still managed by the gastroenterologists for routine monitoring, PCPs such as GPs, pharmacists, and dietitians are involved in the ongoing management of these patients as they require a lifelong elimination of all gluten‐containing grains. In IBD, the complexity of management requires the contribution of multiple healthcare professionals. An ideal IBD team should involve gastroenterologists, surgeons, nurses, dietitians, psychologists, pathologists, radiologists, and pharmacists as outlined in the current Australian IBD Standards.^57^ It is interesting to note that the current Australian IBD Standards do not consider GPs as an integral part of an ideal team.^57^

Although it would be noteworthy to explore why PCPs were not considered important in the care of patients with IBD, none of the research papers identified in this review provided any insights into this phenomenon. Evidence in literature suggests that the lack of PCP importance is because of a lack of training or education regarding the disease as well as suboptimal knowledge and comfort in disease management.^30^, ^71^ This may be as a result of a gap in the provision of supportive education tools for primary care practitioners to provide IBD care. The available guidelines and tools may be helpful, but not suited to primary care practices and are mostly targeted for specialists.^70^ In addition, PCPs care for few IBD patients on a regular basis, which may influence the lack of knowledge and the level of comfort in general with IBD and patients.^71^ Current management of IBD can be optimized through increased knowledge of and familiarity with IBD among PCPs along with accessibility of care for patients. The out‐of‐hospital care for IBD patients that could lead to clinical benefits does not necessarily require gastroenterologists or specialist care, but rather could be provided by IBD nurses and extended roles of PCPs such GPs, pharmacists, psychologists, and other healthcare professionals.^71^ In these instances, the care of patients with IBD would generally pertain to safety monitoring of immunomodulators and biologics, encouraging adherence and compliance, early detection of flares where the patients avoid seeking further medical advice and answering health and lifestyle concerns.^30^, ^71^ This “specialist” model of IBD disease management reveals the absence of a multidisciplinary approach that should include PCPs (GPs and pharmacists). Interestingly, despite research demonstrating the benefits of a multidisciplinary team approach, we found gaps in the provision of adequate and integrated IBD care to patients.^7^, ^58^, ^64^ Ricci *et al*., Lee *et al*., Koltun, and Mikocka‐Walus *et al*. all reported that integrated multidisciplinary models of care in IBD led to improved patient satisfaction and outcomes, a better quality of life and effective health care utilization for both in‐patient and out‐patient management.^20^, ^48^, ^72^, ^73^ It is clear that the benefits of having a multidisciplinary team are multiple, namely, improved continuity of care and reduced associated healthcare costs in the management of patients.^7^, ^8^, ^16^, ^58^ As previously mentioned, and in accordance with other studies, we found roles for, and benefits of, nurses in the management of chronic gastrointestinal diseases such as IBD.^66^, ^68^, ^69^ Although nurses are known to play an integral role in IBD management, this review identified gaps in primary care. The care delivered by nurses is generally provided in secondary/tertiary settings, that is, in hospital as an inpatient or in clinics as an outpatient,^7^, ^58^ and such services are not readily available or accessible in primary care settings, contributing to gaps in the provision of care for IBD patients. This could be optimized if PCPs, such as GPs and pharmacists, were more involved in IBD care.

A lack of understanding and of the available literature has created a sense ambiguity surrounding the roles of PCPs in the management of IBD. We found gaps in the continuity of care between secondary and primary healthcare professionals; for example, issues related to the information provided to patients by healthcare professionals may be contradictory,^41^, ^42^, ^43^, ^74^ there can be a lack of educational tools to assist healthcare professionals in the management of patients with IBD, and there is a paucity of published literature on the primary care management of IBD.^30^, ^71^ In particular, studies showed inconsistent or variable IBD care for patients,^7^, ^58^ knowledge gaps among healthcare professionals,^41^, ^42^, ^43^, ^48^, ^54^, ^56^ a lack of guidelines for primary care practitioners to provide quality of care,^30^, ^48^ and a communication gap between patients and specialists/GPs affecting the delivery of IBD care.^48^, ^51^, ^71^ Interestingly, a contributing factor to gaps in care could be associated with variations in the perceived understanding of disease control between patients and gastroenterologists as outlined in studies by Rubin *et al*. and Holt *et al*. Specialists and clinicians vary in their treatment patterns and recommendations and this can lead to challenging IBD management for patients.^75^, ^76^ Although these demonstrate gaps in care, they also underline the need for suitably tailored guidelines for PCPs, thereby clarifying their roles and enabling them to deliver optimal IBD care. Patients often initially present to their PCPs with symptoms or complications of IBD. PCPs are instrumental in providing not only acute care but also individualized preventive care to IBD patients. Preventative health maintenance is essential for the optimal management of IBD patients^70^, ^77^; however, studies have shown that these patients are at high risk of not receiving maintenance care and/or screening.^8^, ^19^, ^64^ As reported by Andrews *et al*., Bennett *et al*., and a 2013 report *Improving Inflammatory Bowel Disease care across Australia*,^8^, ^30^, ^64^ this can provide opportunities for PCPs to optimize care in key areas in IBD. Examples of this include managing adherence to therapy, monitoring treatment efficacy, smoking cessation, vaccination, screening for cancers (skin, colorectal cervical), and providing education on self‐management.^64^, ^77^ Collaborations involving primary healthcare professionals; GPs and pharmacists; and secondary/tertiary healthcare professionals, nurses, and gastroenterologists can work synergistically toward achieving efficient and improved patient outcomes that could indeed help to significantly reduce both the economic and the clinical burdens of IBD.^16^, ^48^, ^78^

The studies included in this review differed in their design, outcomes, and measurements, and this heterogeneity reduced our ability to make a more precise assessment of key trends. Many of the qualitative studies had only a small sample size and provided merely descriptive information, with limited scope for transferability, and because of this, the results may have been dominated by one group of healthcare professionals. However, in terms of generalizability, while there may indeed be differences in health systems and in practices, the findings from some studies from other countries support those from Australia.

## Conclusion

Multidisciplinary teams provide better care to IBD patients but are rarely implemented in practice. Gaps in care is a research theme that is largely associated with IBD and may be because of the absence of a practical multidisciplinary model of care. Patients with IBD have significant disease‐related complications, which can be present even when the patients may be in remission. PCPs are uniquely placed to facilitate and deliver an integrated multidisciplinary model of care to IBD patients. Despite its limitations, this review has provided a valuable insight into the roles of healthcare professionals in the management of patients with IBD as currently little data exist on the primary care management of IBD. However, further research is still needed to explore opportunities for timely interventions and proactive management, by means of which the economic burden of this disease can potentially be reduced and the care of IBD patients optimised.

## Supporting information


**Table S1** List of the inclusion/exclusion criteria for the review.

**Table S2** List of IBD organizations and society.

**Table S3** Summary of the included studies.

## References

[1] Peery AF , Dellon ES , Lund J *et al* Burden of gastrointestinal disease in the United States: 2012 update. Gastroenterology. 2012; 143: 1179–87.e3. 22885331 10.1053/j.gastro.2012.08.002 PMC3480553

[2] Bernstein CN , Fried M , Krabshuis JH *et al* World Gastroenterology Organization Practice Guidelines for the diagnosis and management of IBD in 2010. Inflamm. Bowel Dis. 2010; 16: 112–24. 19653289 10.1002/ibd.21048

[3] Morrison G , Headon B , Gibson P . Update in inflammatory bowel disease. Aust. Fam. Physician. 2009; 38: 956–61. 20369146

[4] Gastroenterological Society of Australia . *Clinical Update for General Practitioners and Physicians—Inflammatory Bowel Disease [Guideline]*, 2017 [updated 2017. 50]. [Accessed 9 May 2018]. Available from URL: http://cart.gesa.org.au/membes/files/Resources/AIBDA_2017_IBD_Clinical_Update_acknowl_edited.pdf

[5] Jones R . Primary care research and clinical practice: gastroenterology. Postgrad. Med. J. 2008; 84: 454–8. 18940946 10.1136/pgmj.2008.068361

[6] Ng SC , Shi HY , Hamidi N *et al* Worldwide incidence and prevalence of inflammatory bowel disease in the 21st century: a systematic review of population‐based studies. Lancet. 2018; 390: 2769–78. 29050646 10.1016/S0140-6736(17)32448-0

[7] Crohn's Colitis Australia . *Final Report of the First Audit of the Organisation and Provision of IBD Services in Australia 2016 [Report]* 2017 [updated 08/02/2017. 08/02/2017:[98]]. [Accessed 9 May 2018]. Available from URL: https://www.crohnsandcolitis.com.au/site/wp-content/uploads/Final_Web_Audit_17-Updated-3-Feb-2.pdf

[8] PricewaterhouseCoopers (PwC) Australia . *Improving Inflammatory Bowel Disease Care across Australia [Report]* 2013 [updated March, 2013. 53]. [Accessed 9 May 2018]. Available from URL: https://www.crohnsandcolitis.com.au/site/wp-content/uploads/PwC-report-2013.pdf

[9] Botoman VA , Bonner GF , Botoman DA . Management of inflammatory bowel disease. Am. Fam. Physician. 1998; 57: 57–68 71–2. 9447214

[10] Deloitte Access Economics Pty Limited . *The Economic Costs of Crohn's Disease and Ulcerative Colitis [Report]* 2007 [updated 9th June 2007. 100]. [Accessed 9 May 2018]. Available from URL: https://www.crohnsandcolitis.com.au/site/wp-content/uploads/Deloitte-Access-Economics-Report.pdf

[11] Odes S . How expensive is inflammatory bowel disease? A critical analysis. World J. Gastroenterol. 2008; 14: 6641–7. 19034966 10.3748/wjg.14.6641 PMC2773305

[12] Petryszyn PW , Witczak I . Costs in inflammatory bowel diseases. Prz Gastroenterol. 2016; 11: 6–13. 27110304 10.5114/pg.2016.57883 PMC4814543

[13] Bernstein CN , Wajda A , Svenson LW *et al* The epidemiology of inflammatory bowel disease in Canada: a population‐based study. Am. J. Gastroenterol. 2006; 101: 1559–68. 16863561 10.1111/j.1572-0241.2006.00603.x

[14] Molodecky NA , Soon IS , Rabi DM *et al* Increasing incidence and prevalence of the inflammatory bowel diseases with time, based on systematic review. Gastroenterology. 2012; 142: 46–54.e42; quiz e30. 22001864 10.1053/j.gastro.2011.10.001

[15] Pinchbeck BR , Kirdeikis J , Thomson AB . Inflammatory bowel disease in northern Alberta. An epidemiologic study. J. Clin. Gastroenterol. 1988; 10: 505–15. 3263409 10.1097/00004836-198810000-00007

[16] Sack C , Phan VA , Grafton R *et al* A chronic care model significantly decreases costs and healthcare utilisation in patients with inflammatory bowel disease. J. Crohns Colitis. 2012; 6: 302–10. 22405166 10.1016/j.crohns.2011.08.019

[17] Kamm MA . Rapid changes in epidemiology of inflammatory bowel disease. Lancet. 2018; 390: 2741–2. 29050647 10.1016/S0140-6736(17)32669-7

[18] Kaplan GG , Ng SC . Understanding and preventing the global increase of inflammatory bowel disease. Gastroenterology. 2017; 152: 313–21.e2. 27793607 10.1053/j.gastro.2016.10.020

[19] Panes J , O'Connor M , Peyrin‐Biroulet L , Irving P , Petersson J , Colombel JF . Improving quality of care in inflammatory bowel disease: what changes can be made today? J. Crohns Colitis. 2014; 8: 919–26. 24713174 10.1016/j.crohns.2014.02.022

[20] Ricci C , Lanzarotto F , Lanzini A . The multidisciplinary team for management of inflammatory bowel diseases. Dig. Liver Dis. 2008; 40(Suppl. 2): S285–8. 18599002 10.1016/S1590-8658(08)60539-3

[21] Sandborn WJ , Hanauer S , Van Assche G *et al* Treating beyond symptoms with a view to improving patient outcomes in inflammatory bowel diseases. J. Crohns Colitis. 2014; 8: 927–35. 24713173 10.1016/j.crohns.2014.02.021

[22] Krass I , Taylor SJ , Smith C , Armour CL . Impact on medication use and adherence of Australian pharmacists' diabetes care services. J. Am. Pharm. Assoc. (2003). 2005; 45: 33–40. 15730115 10.1331/1544345052843093

[23] Emmerton LM , Smith L , LeMay KS *et al* Experiences of community pharmacists involved in the delivery of a specialist asthma service in Australia. BMC Health Serv. Res. 2012; 12: 164. 22709371 10.1186/1472-6963-12-164 PMC3439711

[24] Butt M , Mhd Ali A , Bakry MM , Mustafa N . Impact of a pharmacist led diabetes mellitus intervention on HbA1c, medication adherence and quality of life: A randomised controlled study. Saudi Pharm. J. 2016; 24: 40–8. 26903767 10.1016/j.jsps.2015.02.023 PMC4720029

[25] Codispoti C , Douglas MR , McCallister T , Zuniga A . The use of a multidisciplinary team care approach to improve glycemic control and quality of life by the prevention of complications among diabetic patients. J. Okla. State Med. Assoc. 2004; 97: 201–4. 15212108

[26] Jack L Jr , Airhihenbuwa CO , Namageyo‐Funa A , Owens MD , Vinicor F . The psychosocial aspects of diabetes care. Using collaborative care to manage older adults with diabetes. Geriatrics. 2004; 59: 26–31 quiz 2. 15152733

[27] Renders CM , Valk GD , Griffin S , Wagner EH , Eijk JT , Assendelft WJ . Interventions to improve the management of diabetes mellitus in primary care, outpatient and community settings. Cochrane Database Syst. Rev. 2001; 1: CD001481. 10.1002/14651858.CD001481 PMC7045779 11279717

[28] Wagner EH , Glasgow RE , Davis C *et al* Quality improvement in chronic illness care: a collaborative approach. Jt. Comm. J. Qual. Improv. 2001; 27: 63–80. 11221012 10.1016/s1070-3241(01)27007-2

[29] Wagner EH , Sandhu N , Newton KM , McCulloch DK , Ramsey SD , Grothaus LC . Effect of improved glycemic control on health care costs and utilization. JAMA. 2001; 285: 182–9. 11176811 10.1001/jama.285.2.182

[30] Bennett AL , Munkholm P , Andrews JM . Tools for primary care management of inflammatory bowel disease: do they exist? World J. Gastroenterol. 2015; 21: 4457–65. 25914455 10.3748/wjg.v21.i15.4457 PMC4402293

[31] Liberati A , Altman DG , Tetzlaff J *et al* The PRISMA statement for reporting systematic reviews and meta‐analyses of studies that evaluate health care interventions: explanation and elaboration. Ann. Intern. Med. 2009; 151: W65–94. 19622512 10.7326/0003-4819-151-4-200908180-00136

[32] Moher D , Liberati A , Tetzlaff J , Altman DG , Group P . Preferred reporting items for systematic reviews and meta‐analyses: the PRISMA statement. Ann. Intern. Med. 2009; 151: 264–9 W64. 19622511 10.7326/0003-4819-151-4-200908180-00135

[33] Nagpal K , Vats A , Lamb B *et al* Information transfer and communication in surgery: a systematic review. Ann. Surg. 2010; 252: 225–39. 20647929 10.1097/SLA.0b013e3181e495c2

[34] Turner‐Stokes L , Harding R , Sergeant J , Lupton C , McPherson K . Generating the evidence base for the National Service Framework for Long Term Conditions: a new research typology. Clin. Med. (Lond.). 2006; 6: 91–7. 16521364 10.7861/clinmedicine.6-1-91 PMC4954443

[35] Mayer H , Nonn C , Osterbrink J , Evers GC . Quality criteria of assessment scales—Cohen's kappa as measure of interrator reliability (1). Pflege. 2004; 17: 36–46. 15040245 10.1024/1012-5302.17.1.36

[36] McHugh ML . Interrater reliability: the kappa statistic. Biochem. Med. (Zagreb). 2012; 22: 276–82. 23092060 PMC3900052

[37] Bebb JR , Lawson A , Knight T , Long RG . Long‐term follow‐up of coeliac disease‐‐what do coeliac patients want? Aliment. Pharmacol. Ther. 2006; 23: 827–31. 16556185 10.1111/j.1365-2036.2006.02824.x

[38] Bengtsson M , Ulander K , Borgdal EB , Christensson AC , Ohlsson B . A course of instruction for women with irritable bowel syndrome. Patient Educ. Couns. 2006; 62: 118–25. 16098703 10.1016/j.pec.2005.06.015

[39] Bengtsson M , Ulander K , Borgdal EB , Ohlsson B . A holistic approach for planning care of patients with irritable bowel syndrome. Gastroenterol. Nurs. 2010; 33: 98–108. 20389223 10.1097/SGA.0b013e3181d60026

[40] Bremner H . Nurse‐led hypnotherapy: an innovative approach to Irritable Bowel Syndrome. Complement. Ther. Clin. Pract. 2013; 19: 147–52. 23890461 10.1016/j.ctcp.2013.01.001

[41] Czuber‐Dochan W , Norton C , Bredin F , Darvell M , Nathan I , Terry H . Healthcare professionals' perceptions of fatigue experienced by people with IBD. J. Crohns Colitis. 2014; 8: 835–44. 24491516 10.1016/j.crohns.2014.01.004

[42] Dickman R , Segev M , Levi S *et al* Perceptions of gastroenterologists and nurses regarding irritable bowel syndrome and inflammatory bowel disease. Eur. J. Gastroenterol. Hepatol. 2011; 23: 813–7. 21701390 10.1097/MEG.0b013e328348a552

[43] Dupuis M , Marshall JK , Hayes SM , Cytryn K , Murray S . Assessing the educational needs of Canadian gastroenterologists and gastroenterology nurses: challenges to optimal care in Crohn's Disease. Can. J. Gastroenterol. 2009; 23: 805–10. 20011732 10.1155/2009/384926 PMC2805516

[44] Gerson CD , Gerson MJ . A collaborative health care model for the treatment of irritable bowel syndrome. Clin. Gastroenterol. Hepatol. 2003; 1: 446–52. 15017644 10.1016/s1542-3565(03)00218-0

[45] Krishnan HS , Schaefer M . Evaluation of the impact of pharmacist's advice giving on the outcomes of self‐medication in patients suffering from dyspepsia. Pharm. World Sci. 2000; 22: 102–8. 11028264 10.1023/a:1008733207854

[46] Leach P , De Silva M , Mountifield R *et al* The effect of an inflammatory bowel disease nurse position on service delivery. J. Crohns Colitis. 2014; 8: 370–4. 24161810 10.1016/j.crohns.2013.09.018

[47] Mehuys E , Van Bortel L , De Bolle L , Van Tongelen I , Remon JP , De Looze D . Self‐medication of upper gastrointestinal symptoms: a community pharmacy study. Ann. Pharmacother. 2009; 43: 890–8. 19417113 10.1345/aph.1L647

[48] Mikocka‐Walus A , Andrews JM , Rampton D , Goodhand J , van der Woude J , Bernstein CN . How can we improve models of care in inflammatory bowel disease? An international survey of IBD health professionals. J. Crohns Colitis. 2014; 8: 1668–74. 25132216 10.1016/j.crohns.2014.07.009

[49] Ringstrom G , Storsrud S , Simren M . A comparison of a short nurse‐based and a long multidisciplinary version of structured patient education in irritable bowel syndrome. Eur. J. Gastroenterol. Hepatol. 2012; 24: 950–7. 22617366 10.1097/MEG.0b013e328354f41f

[50] Roberts L , Wilson S , Singh S , Roalfe A , Greenfield S . Gut‐directed hypnotherapy for irritable bowel syndrome: piloting a primary care‐based randomised controlled trial. Br. J. Gen. Pract. 2006; 56: 115–21. 16464325 PMC1828217

[51] Stretton JG , Currie BK , Chauhan UK . Inflammatory bowel disease nurses in Canada: an examination of Canadian gastroenterology nurses and their role in inflammatory bowel disease care. Can. J. Gastroenterol. Hepatol. 2014; 28: 89–93. 24501725 10.1155/2014/179309 PMC4071889

[52] Teichert M , Griens F , Buijs E , Wensing M , De Smet PA . Effectiveness of interventions by community pharmacists to reduce risk of gastrointestinal side effects in nonselective nonsteroidal anti‐inflammatory drug users. Pharmacoepidemiol. Drug Saf. 2014; 23: 382–9. 24535837 10.1002/pds.3587

[53] Urwin H , Wright D , Twigg M , McGough N . Early recognition of coeliac disease through community pharmacies: a proof of concept study. Int. J. Clin. Pharmacol. 2016; 38: 1294–300. 10.1007/s11096-016-0368-4 PMC5031749 27503280

[54] Casellas F , Marin‐Jimenez I , Borruel N , Riestra S . Ulcerative colitis in remission: How to improve adherence from a a multidisciplinary perspective. ScienceDirect. 2016; 15: 37–43.

[55] Reid LW , Chivers S , Plummer V , Gibson P . Inflammatory bowel disease management: a review of nurses' role in Australia and the United Kingdom. Aust. J. Adv. Nurs. 2009; 27: 19–26.

[56] Bager P , Hentze R , Nairn C . Outpatients with inflammatory bowel disease (IBD) strongly prefer annual telephone calls from an IBD nurse instead of outpatient visits. Gastroenterol. Nurs. 2013; 36: 92–6. 23549211 10.1097/SGA.0b013e318288c8a8

[57] Crohn's Colitis Australia (CCA) . *Australian IBD Standards: Standards of Healthcare for People with Inflammatory Bowel Disease in Australia [Guideline]* 2016 [2015—interim standards 16]. [Accessed 9 May 2018]. Available from URL: https://www.crohnsandcolitis.com.au/site/wp-content/uploads/IBD-Standards-Final.pdf

[58] Royal College of Physicians UK . *A summary of the UK Inflammatory Bowel Disease Audit 2014 [Report]* 2014 [updated 2014. 28]. [Accessed 9 May 2018]. Available from URL: https://www.rcplondon.ac.uk/file/2441/download?token=aBn5fr4g

[59] IBD Standards Group . *IBD Standards 2013 Update—Standards for the Healthcare of People Who Have Inflammatory Bowel Disease (IBD). [Guideline]* 2013 [updated 2013]. [Accessed 10 August 2018]. Available from URL: http://s3-eu-west-1.amazonaws.com/files.crohnsandcolitis.org.uk/Publications/PPR/ibd-standards.pdf

[60] National Institute of Health and Care Excellence (NICE) . *Inflammatory Bowel Disease—Quality Standards [Guidelines]* 2015 [26 Feb 2015:[39]. [Accessed 8 August 2018]. Available from URL: https://www.nice.org.uk/guidance/qs81/resources/inflammatory-bowel-disease-pdf-2098903535557

[61] Belling R , Woods L , McLaren S . Stakeholder perceptions of specialist Inflammatory Bowel Disease nurses' role and personal attributes. Int. J. Nurs. Pract. 2008; 14: 67–73. 18190486 10.1111/j.1440-172X.2007.00661.x

[62] Casellas F , Vera I , Ginard D , Torrejon A . Grupo Espanol de Trabajo En Enfermedad de Crohn YCUG. Inflammatory bowel disease patient's satisfaction with healthcare services received. Physicians' and nurses' perceptions. Rev. Esp. Enferm. Dig. 2013; 105: 385–91. 24206548 10.4321/s1130-01082013000700003

[63] Clement C , Rapport F , Seagrove A , Alrubaiy L , Williams J . Healthcare professionals' views of the use and administration of two salvage therapy drugs for acute ulcerative colitis: a nested qualitative study within the CONSTRUCT trial. BMJ Open. 2017; 7: e014512. 10.1136/bmjopen-2016-014512 PMC5337666 28399515

[64] Andrews JM , Mountifield RE , Van Langenberg DR , Bampton PA , Holtmann GJ . Un‐promoted issues in inflammatory bowel disease: opportunities to optimize care. Intern. Med. J. 2010; 40: 173–82. 19849744 10.1111/j.1445-5994.2009.02110.x

[65] Gikas A , Triantafillidis JK . The role of primary care physicians in early diagnosis and treatment of chronic gastrointestinal diseases. Int. J. Gen. Med. 2014; 7: 159–73. 24648750 10.2147/IJGM.S58888 PMC3958525

[66] Hernandez‐Sampelayo P , Seoane M , Oltra L *et al* Contribution of nurses to the quality of care in management of inflammatory bowel disease: a synthesis of the evidence. J. Crohns Colitis. 2010; 4: 611–22. 21122570 10.1016/j.crohns.2010.08.009

[67] Royal College of Nursing . *Roles Descriptives for Inflammatory Bowel Disease—Nurse Specialists: RCN Guidance [Guideline]* [updated 2007. 18]. 2007 [Accessed 8 August 2018]. Available from URL: https://www.rcn.org.uk/professional-development/publications/pub-003194

[68] Younge L , Norton C . Contribution of specialist nurses in managing patients with IBD. Br. J. Nurs. 2007; 16: 208–12. 17363850 10.12968/bjon.2007.16.4.22979

[69] O'Connor M , Bager P , Duncan J *et al* N‐ECCO Consensus statements on the European nursing roles in caring for patients with Crohn's disease or ulcerative colitis. J. Crohns Colitis. 2013; 7: 744–64. 23831217 10.1016/j.crohns.2013.06.004

[70] Gastroenterological Society of Australia . *Inflammatory Bowel Disease Clinical Update Fourth Edition Updated 2018 [Guideline]* 2018 [42]. [Accessed 8 September 2018]. Available from URL: http://cart.gesa.org.au/membes/files/Resources/2018_IBD_Clinical_Update_May_update.pdf

[71] Tan M , Holloway RH , Lange K , Andrews JM . General practitioners' knowledge of and attitudes to inflammatory bowel disease. Intern. Med. J. 2012; 42: 801–7. 21883783 10.1111/j.1445-5994.2011.02586.x

[72] Koltun WA . Better together: improved care of the IBD patient using the multi‐disciplinary IBD center. Expert Rev. Gastroenterol. Hepatol. 2017; 11: 491–3. 28317406 10.1080/17474124.2017.1309289

[73] Lee CK , Melmed GY . Multidisciplinary team‐based approaches to IBD management: how might "one‐stop shopping" work for complex IBD care? Am. J. Gastroenterol. 2017; 112: 825–7. 28508869 10.1038/ajg.2017.124

[74] Singh S , Chowdhry M , Umar S , Bilal M , Clarke K . Variations in the medical treatment of inflammatory bowel disease among gastroenterologists. Gastroenterol. Rep. (Oxf.). 2018; 6: 61–4. 29479445 10.1093/gastro/gox005 PMC5806403

[75] Holt DQ , Strauss BJ , Moore GT . Patients with inflammatory bowel disease and their treating clinicians have different views regarding diet. J. Hum. Nutr. Diet. 2017; 30: 66–72. 27412965 10.1111/jhn.12400

[76] Rubin DT , Dubinsky MC , Martino S , Hewett KA , Panes J . Communication Between Physicians and Patients with Ulcerative Colitis: Reflections and Insights from a Qualitative Study of In‐Office Patient‐Physician Visits. Inflamm. Bowel Dis. 2017; 23: 494–501. 28296817 10.1097/MIB.0000000000001048 PMC5495553

[77] Abegunde AT , Muhammad BH , Ali T . Preventive health measures in inflammatory bowel disease. World J. Gastroenterol. 2016; 22: 7625–44. 27678347 10.3748/wjg.v22.i34.7625 PMC5016364

[78] Mikocka‐Walus A , Andrews JM , von Kanel R , Moser G . An improved model of care for inflammatory bowel disease (IBD). J. Crohns Colitis. 2013; 7: e120–1. 22959397 10.1016/j.crohns.2012.08.004

