# Supplementary material for: Roles of healthcare professionals in the management of chronic gastrointestinal diseases with a focus on primary care: A systematic review
Source: JGH Open. 2019 Aug 27;4(2):221–9. doi: 10.1002/jgh3.12235 (PMC7144774; doi:10.1002/jgh3.12235)
Supplement: Supplementary file 1 — Table S1 List of the inclusion/exclusion criteria for the review. [file JGH3-4-221-s001.docx]

**Supporting Information**

Table 1 List of the Inclusion/Exclusion Criteria for the Review

| Inclusion Criteria |
| --- |
| English language |
| Date restrictions – 1970 onwards |
| GI Diseases (GID) – IBD; IBS; FD/dyspepsia; Coeliac/Celiac |
| Adults (age 18 years or older) |
| Primary research studies |
| Primary/community care setting/general practice |
| Model of Care; Disease Management involving healthcare professionals e.g. patient perception, allied health perception |
| Any domain in relation to GI Disease e.g. QOL |
| Studies looking at gaps in management of GI Disease |
| Exclusion Criteria |
| Reviews, discussion papers, conference article, comments, notes etc. |
| No full text available |
| Only secondary/tertiary setting |
| No mention of sample size/control populations/comparison groups. |
