# Supplementary material for: Roles of healthcare professionals in the management of chronic gastrointestinal diseases with a focus on primary care: A systematic review
Source: JGH Open. 2019 Aug 27;4(2):221–9. doi: 10.1002/jgh3.12235 (PMC7144774; doi:10.1002/jgh3.12235)
Supplement: Supplementary file 2 — Table S2 List of IBD organizations and society. [file JGH3-4-221-s002.docx]

**Supporting Information:**

Table 2 List of IBD Organisations and Society

| **IBD Organisation/Society** | **Website** |
| --- | --- |
| Crohn’s & Colitis Australia (CCA) | [www.cronhsandcolitis.com.au](http://www.cronhsandcolitis.com.au) |
| Crohn’s & Colitis Foundation | [www.crohnscolitisfoundation.org](http://www.crohnscolitisfoundation.org) |
| Crohn’s and Colitis Canada | [www.crohnsandcolitis.ca](http://www.crohnsandcolitis.ca) |
| Crohn’s & Colitis UK | [www.crohnsandcolitis.org.uk](http://www.crohnsandcolitis.org.uk) |
| European Federation of Crohn’s and Ulcerative Colitis Associations (EFCCA) | [www.efcca.org](http://www.efcca.org) |
| Irish Society for Colitis and Crohn’s Disease (ISCC) | [www.iscc.ie](http://www.iscc.ie) |
| British Society of Gastroenterology | [www.bsg.org.uk](http://www.bsg.org.uk) |
| Gastroenterological Society of Australia | [www.gesa.org.au](http://www.gesa.org.au) |
| The Digestive Disorders Foundation | [www.digestivedisorders.org.uk](http://www.digestivedisorders.org.uk) |
| National Association for Colitis and Crohn’s Disease | [www.nacc.org.uk](http://www.nacc.org.uk) |
| The National Institute for Health and Care Excellence (NICE) | [www.nice.org.uk](http://www.nice.org.uk) |
| Royal College of Physicians of London (GB) | [www.rcplondon.ac.uk](http://www.rcplondon.ac.uk) |
