# Supplementary material for: Roles of healthcare professionals in the management of chronic gastrointestinal diseases with a focus on primary care: A systematic review
Source: JGH Open. 2019 Aug 27;4(2):221–9. doi: 10.1002/jgh3.12235 (PMC7144774; doi:10.1002/jgh3.12235)
Supplement: Supplementary file 3 — Table S3 Summary of the included studies. [file JGH3-4-221-s003.docx]

**Supporting Information**

Table 3 Summary of the included studies

| **Reference (first author/year)** | **Country** | **Study methodology** | **Healthcare Professionals (HCP)** | **Key findings** | **Level of evidence/comments** |
| --- | --- | --- | --- | --- | --- |
| Descriptive Studies (Qualitative) | | | | | |
| Casellas et al. 2016 ⁵⁸ | Spain | Used a means of discussion focus groups among 4 primary care physicians, 4 gastroenterologists and 4 nurses who interact with patients with ulcerative colitis: 3 individual and 1 mixed. | Primary care physicians (PCP), Gastroenterologists (GE), gastroenterology (GI) nurses | PCP gave less importance to adherence vs. nurses & GE. This describes a fundamental aspect to possibly improving adherence in patients through simple yet effective measures. As well as a gap in knowledge and understanding of managing patients with UC. | 9/20: Qualitative research has several weakness - details of meeting, framework and focus group not mentioned along with a small sample size. |
| Czuber-Dochan et al. 2014 ⁴⁵ | UK | Phenomenology study conducted with 20 HCPs working with IBD patients. In-depth semi-structured interviews were audio recorded and transcribed verbatim. | HPCs - GE, nurses, dietitians, GP, hospital pharmacists, psychologist, counsellor, psychiatrist | HCPs have varied level of knowledge and awareness of IBD fatigue; participants had problems with understanding and reporting fatigue. This study identified a gap in HCP's knowledge and understanding of the complexity of IBD fatigue and its full impact on patients’ lives. | 12/20: Small sample size is not a numeric representation of the studied population and may be dominated by one group of HCP e.g. 8 IBD nurses vs. 2 GPs/pharmacists. |
| Mixed Method Studies | | | | | |
| Dupuis et al. 2009 ⁴⁷ | Canada | 213 GE, 36 nurses and a sample of patients used to determine the nature and extent of knowledge gaps and barriers in care of CD patients. | GE, GI nurses | 46 GE & 40 GI nurses - lacked clarity regarding their roles and responsibilities across continuum of care. Self-identified gaps in skills, knowledge, confidence in monitoring disease progression, effectively assessing response to therapy & patient communication and education to enhance adherence to treatment. | 13/20: Only assessed clinical practice, barriers to change and knowledge gaps and areas of adequate care reported were not addressed. |
| Krishnan et al. 2000 ⁴⁹ | Germany | Randomised allocation to study or control group to assess the outcome of self-medication and demonstrate the value of pharmacist involvement in the process. | Pharmacists | Patients from the intervention pharmacies had a lower HrQOL than patients of control pharmacies before pharmacist intervention. Pharmacist counselling had a positive impact on self-medication outcomes. | 12/20: self-reporting bias; drug-related issues in dyspepsia was not sufficiently assessed. |
| Urwin et al. 2016 ⁵⁷ | UK | Looking at POCT testing as a targeted case finding service for coeliac disease by pharmacists/pharmacy. | Pharmacists | 551 individuals tested for coeliac with 9.4% (52) tested positive; most common presenting problem for recruitment was IBS symptoms (50.3%) with 25% for diarrhoea. POCT was effective in early recognition of coeliac disease; pharmacists can successfully deliver such service to patients. | 14/20: details on the profile of respondents and analysis are not specified and self-selection bias with pharmacist interviews. |
| Descriptive Studies (Quantitative) | | | | | |
| Bager et al. 2013 ⁶⁰ | Denmark | 150 participated in a questionnaire survey - using 5 Likert scale, patients’ perception on willingness and concerns to changing regular visits to annual telephone call from an IBD nurse. | Nurses | 147 patients responded - 87% agreeing "100% with SM approach" or agreeing with some concerns; 25% of patients had concerns or comments. Patients were agreeable to changing to a telephone call method as it was most convenient. | 15/20: Possible bias in patient selection. |
| Bebb et al. 2006 ⁴¹ | UK | 183 patients assessed on their views on long term management of coeliac disease through 10-point questionnaire focused on dietary practice and follow-up method. | GE, dietitians, GP | 126 patients completed questionnaires - Patients prefer to see a dietitian with a possible option of a doctor available, prefer annual review follow up and ; no major comparable difference between those diagnosed more recently (5yrs) vs those >5yrs. | 14/20: limited questionnaire details (structure and reliability). |
| Dickman et al. 2011 ⁴⁶ | Israel | IPQ-R adapted for medical staff was sent randomly to 300 physicians and nurses to assess their perceptions of IBD and IBS and were selected from hospital and community. | GI physicians, nurses | Only 33% completed questionnaire - 150 = IBD & 150 = IBS; more from hospital (76) than community (15). Overall, Physicians and nurses have different attitudes to and perceptions of IBD and IBS in terms of chronicity, severity, treatment efficacy, personal control and understanding of the illness. | 12/20: Low response rate of 32% from community professionals; comparison between GI physicians and nurses may be masked by effect of sex. |
| Mehuys et al. 2009 ⁵¹ | Belgium | 63 community pharmacies assessed 592 patients’ self-medication behaviour and adherence to pharmacist advice. | Pharmacists | Most common reported GI symptoms were burning retrosternal discomfort (49%), acid regurgitation (53%), postprandial fullness (51%); 51% of patients consulted their GP upon pharmacist advice. Almost all participants who were advised on self-treatment reported symptom relief with OTC drug. | 16/20: recruited sample is not representative of all individuals seeking self-treatment for GI symptoms; does not take into account chronic GI condition sufferers. |
| Stretton et al. 2014 ⁵⁵ | Canada | 29-questions survey used to investigate IBD nursing role and services provided across Canada of 275 nurses. | Nurses | 98% are female nurses, where 43% work in endoscopy unit and only 30% had a primary role in IBD; majority worked with adult IBD patients and few in paediatrics. Very small percentage of nurses provide clinical IBD care and had multiple roles/responsibilities providing variety of non IBD services. | 15/20: response rate not calculated, due to unclear distribution numbers. Lack of definition of nurse classification for selection. |
| Intervention Studies | | | | | |
| Bengtsson et al. 2006 ⁴² | Sweden | 29 women with IBS participated in programme of instructions relating to IBS management before and after the course with a 12-month follow-up. | Physician (GE), nurses, dietitian, social worker | 23 women completed the questionnaires at 12mth follow-up; there improvement in values to baseline in abdominal pain, vitality, number of physician visits & dietitian. It shows that multidisciplinary team (MDT) approach improves patient related outcomes in IBS management. | 14/20: no randomization with no control group therefore subject to several sources of bias. |
| Bremner 2013 ⁴⁴ | UK | Assessed effectiveness of nurse-led hypnotherapy in 268 patients at baseline, mid-point and at discharge. Patients received tailored hypnotherapeutic techniques and materials aimed at individual patient's specific needs. | Nurses | Symptom severity: improved in 85% of subjects (30.6 to 17.2); IBS-QOL questionnaire showed marked improvement (from 44.4% to 70.2%); SF-36 questionnaire showed improvement as well. Overall, nurse-led hypnotherapy showed successful results in patient outcomes for IBS. | 11/20: The research questions/aims and objectives are not stated; while the sample size is adequate, statistical analysis sample sizes were too small for different aspects of analysis. |
| Leach et al. 2013 ⁵⁰ | Australia | Measuring the effectiveness of an IBD nurse performed activity in patients by assessing effect on patient management and efficiency along with cost effectiveness analysis. | IBD nurse | 4920 occasions of service (OOS) recorded for 12 month period. IBD nurse interventions led to avoidance of 27 hospital admissions, 32 ED presentations, 163 outpatient reviews and cost saving of AUS$136,535; CD patients utilised nurses more than UC. | 11/20: based around self-reporting of IBD nurse's activity. Does not look at adherence, monitoring of medications, counselling and development of patient centred action plans. |
| Ringstrom et al. 2012 ⁵³ | Sweden | 80 patients randomised based on Rome II criteria to compare effects of short (nurse) vs. long MDT education intervention in IBS along with patient satisfaction. | HCP - GE, nurses, psychologists, dietitians, physiotherapists | 80 patients randomised into 2 groups of 6 session (long MDT) or 3 sessions (short nurse-led). 24 patients were classified as moderate IBS and 49 with severe IBS; no difference in effect on measured outcomes. Both MDT and nurse-led interventions can improve patient outcomes. | 19/20: lack of blinding but impossible to achieve in a non-pharmacological interventions. |
| Randomised Control Trial (RCT) Studies | | | | | |
| Gerson et al. 2003 ⁴⁸ | USA | 41 patients randomly assigned into 3 treatment groups to compare collaborative treatment with medical treatment to relieve symptoms in IBS. | GE, Psychologist | 16 patients completed collaborative treatment model, 8 for medical treatment and 6 for psychological treatment. Collaborative model had significant improvement in 'intent to treat' & per protocol, global self-assessment, major symptoms. | 17/20: used Rome criteria I; whereas now there is IV (possible irrelevance - creating bias). |
| Roberts et al. 2006 ⁵⁴ | UK | 81 patients were divided into intervention and control group and assessed the effectiveness of gut-directed hypnotherapy at baseline and again 3 monthly intervals till 1yr post randomisation. | GE, GP, hypnotherapists | Baseline symptoms and QOL scores were comparable in both groups with difference noted in QOL scores; response rate of 82% for 3 month assessment, 83% for 6mths & 65% for follow-up; intervention group had significant improvement in symptom control (pain, diarrhoea) at 3 months vs. control but no difference maintained over time. | 19/20: Not a definitive trial of hypnotherapy; lack of 3rd arm; incomplete follow-up bias symptom & QOL scores. |
| Cross-sectional Study | | | | | |
| Mikocka-Walus et al. 2014 ⁵² | 10 Countries – UK, Australia, Netherlands, USA, New Zealand, Canada, Italy, Switzerland, France, Ireland. | Recruited 135 HCPs caring for IBD patients to gather information on current care models and their views on reshaping it. | HCP - GE, nurses, psychologists, dietitians, surgeons, psychiatrists, physiotherapists | 50% of participants were GI physicians, 34% nurses, 8% psychologists, 4% dietitians, 2% surgeons and 1% psychiatrists & physiotherapist; 54% participants believed their IBD service had integrated model of care. HCP believe an ideal IBD service should be fully integrated with MDT. | 12/20: unknown response rate; probable participation bias (51% GI physicians); social desirability bias. |
| Pilot Study | | | | | |
| Reid et al. 2009 ⁵⁹ | Australia, UK | 12 Australian and 19 UK nurses to explore the roles of nurses in the provision of IBD health services. | Nurses | Response rate for Australian nurses was 31% and UK nurses 54%; UK nurses were more specialised in IBD & Australian nurses more in clinical trials; UK nurses have dedicated services specific to IBD whilst Australian nurses cater for wider range of conditions. | 7/20: Small sample size plus targeted a specific group. No definition of IBD nurse; data to support the findings are not presented. |
| Cohort Study | | | | | |
| Teichert et al. 2014 ⁵⁶ | Netherlands | Assessing pharmacists engaged in defined protocol with activities targeting interventions of addition of gastro protection/cessation of NSAID use. | Pharmacists | 3 groups (intervention group, control group & unselected patient group); baseline showed 14% of NSAID users at UGI risk did not receive GPAs; additional 7% risk reduction for intervention group at follow-up. Pharmacist-led interventions can improve risk reduction. | 16/20: did not look at adherence/compliance issues with medication safety issues associated with NSAID use. |
| Observational Studies | | | | | |
| UK IBD Audit Steering Group 2014 ⁶² | UK | Assessed the level of compliance/quality of care that IBD patients receive against national standards. | MDT | 14% of sites had no IBD specialist nurse. Of the 70% of sites, 30% had an ideal IBD team; 23% had no access to a dietitian & 91% had IBD nurse specialist telephone access. MDT team approach improves IBD care in patients with IBD specialists nurse playing an important role in IBD management. | 14/20: the report presents key findings and recommendations for each standard. |
| Australian IBD Audit 2016 ¹³ | Australia | Assessed the level of compliance/quality of care that IBD patients receive against national standards. | MDT | IBD care was variable across sites - 1 had a full MDT IBD team and less 25% offered partial IBD service; 39% had IBD nurse provision, 51% had an IBD helpline & less 5% had any psychological support. MDT team with access to specialised services delivered better health outcomes and had reduced admission rates. | 14/20: the report presents key findings and recommendations for each standard. |

*(HCP, healthcare professionals; PCP, primary care physicians; GE, gastroenterologists; GI, gastroenterology; GP, general practitioners; MDT, multi-disciplinary team)*
